# Discovery of genome-wideSNPs by RAD-seqand the genetic diversity of captive hog deer (*Axis porcinus*)

**DOI:** 10.1371/journal.pone.0174299

**Published:** 2017-03-21

**Authors:** Wei Wang, Huijuan Yan, Jianqiu Yu, Jun Yi, Yu Qu, Maozhong Fu, Ang Chen, Hui Tang, Lili Niu

**Affiliations:** 1 Sichuan Animal Science Academy, Chengdu, China; 2 Chengdu Zoo, Chengdu, China; National Cheng Kung University, TAIWAN

## Abstract

The hog deer (*Axis porcinus*) is a small deer whose natural habitat is the wet or moist tall grasslands in South and Southeast Asia. Wild populations have dramatically decreased in recent decades. While wild hog deer were recently acknowledged to be extinct in China, a few captive populations have been maintained. In the present study, we successfully employed the restriction-site-associated DNA sequencing (RAD-seq) technique to generate a genome-wide profile of single-nucleotide polymorphisms (SNPs) in the captive population of hog deer from Chengdu Zoo, China (N = 11). Up to 4.7 million clean reads per sample were sequenced, and 11,155 SNPs and 8,247 haplotypes were simultaneously observed within more than 80% of sequenced individuals. In this population, the mean frequency of major alleles at each polymorphism site was 0.7903±0.0014, and the average nucleotide diversity (π) and inbreeding coefficient (F_IS_) were 0.3031±0.0015 and -0.0302±0.0062, respectively. Additionally, the Euclidean distance-based multidimensional scaling method revealed that the pairwise genetic relatedness was evenly distributed. However, the results of homologous searching by short reads did not provide any meaningful explanation of the phylogenetic relationship of hog deer, which should be further investigated. In conclusion, our results revealed current state of genetic diversity in this captive population of hog deer.Furthermore, these genome-wide SNPs would be useful for guiding the mating schedule to avoid sharp increase of inbreeding coefficient.

## Introduction

The hog deer (*Axis porcinus*) is an endemic species of South and Southeast Asia and can be divided into the Southeast Asian subspecies (*A*.*p*.*annamiticus*) from China, Thailand, Laos, Cambodia and Vietnam, and the Indian subspecies (*A*.*p*.*porcinus*) distributed in Pakistan, Nepal, India, Bangladesh and Burma[1]. The hog deer has a karyotype of 2n = 68 and belongs to the Cervinae subfamily according to genetic information from both mitochondrial and nuclear DNA [2].However, the genus in which hog deer should be phylogenetically positioned is still controversial[3]. Such debates will be better resolved with the increasing availability of molecular and archaeological evidence.

Possibly because of its narrow habitat or other unknown factors, the wild population of hog deer has undergone a serious decline for decades; therefore, the hog deer has been included in the Red List at the Endangered level by the International Union for the Conservation of Nature(IUCN) since 2008 [4]. The historical record of wild hog deer in China was mainly found in Gengma and Cangyuan counties of Western Yunnan; however, the wild population is currently believed to be almost completely eliminated [5,6].Fortunately, there are still a few captive hog deer in China, most of which have been reared in Chengdu Zoo, Sichuan. The phenotypic growth characteristics and physiological indices of captive hog deer from Chengdu Zoo have been specifically investigated[7,8]. However, the gene pool and diversity of this captive population remain largely unknown. Further knowledge is essential to develop an efficient conservation program.

Along with rapid advancements in high-throughput sequencing techniques, genotype-by-sequencing techniques, such as restriction-site-associated DNA sequencing (RAD-seq), provide cost-efficient methods to investigate the genome-wide variants in non-model species even when the reference genome is unavailable[9,10]. The RAD-seq technique was first proposed in 2008 and is mainly characterized by the inclusion of restriction enzyme(s) to randomly digest genomic DNA into small fragments for sequencing[11]. Due to the genome-wide distribution of these sequenced short reads and the high-through put capacity, the RAD-seq technique has been widely used in studies of population genetics and ecology [12].In the present study, we generated a representative profiling of genome-wide single-nucleotide polymorphisms (SNPs) of captive hog deer from Chengdu Zoo using the RAD-seq technique and further investigated their genetic diversity. The results are expected to provide accurate information on the actual genetic structure of this captive population, and hence help us to develop efficient mating schedule to avoid sharp increase of inbreeding coefficient because only a small population is being kept.

## Materials and methods

### Sampling and extraction of genomic DNA

**Fig 1:**
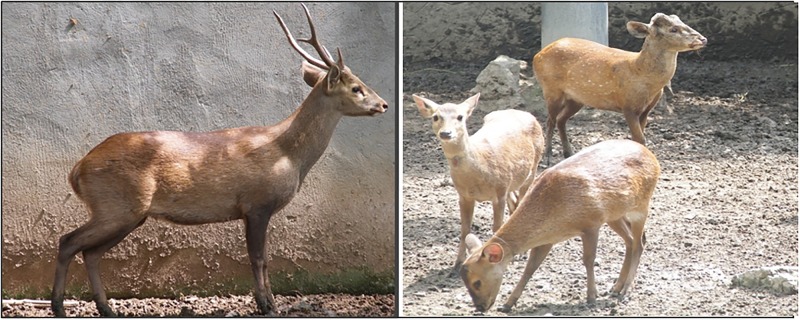
Photos of a male hog deer, approximately five years old (left), and three related individuals (right) reared in Chengdu Zoo.

A total of 11 hog deer(five males and six females)were sampled from the captive population reared in Chengdu Zoo, Chengdu, China (Fig 1). Although the exact mating records are unavailable, we also tried to guarantee that these sampled individuals were as unrelated as possible according to the breeder's subjective recommendation. For example, if multiple individuals were already known to be directly related, such as parent-child and sister relationships, only one of the deer was ultimately sampled.Venous whole blood was collected in the absence of anticoagulants. Genomic DNA was extracted and purified from blood tissue samples according to the protocol of the Animal Genomic DNA Kit (Tiangen, Beijing).NanoVue Plus (GE,USA) was used to assess the DNA concentration and quality.

### RAD sequencing and quality filtering

In the present study, we successfully selected the restriction enzyme *EcoRI* (NEB, Beijing) to digest the genomic DNA (approximately 1 μg per sample) according to our preliminary investigation. The RAD sequencing library was constructed using the recommended pipeline [11].Briefly, the P1 Adaptor sequence was first added to these digested fragments, followed by the sequential steps of sample pooling, random shearing and the addition of the P2 Adaptor sequence. Finally, DNA fragments 300 to 700 bp in length were selected and subjected to sequencing on an Illumina HiSeq^™^ 2000 platform to generate125-bp paired-end reads (Novogene Co. Ltd., Beijing).

The initial images from the sequencer were first converted into sequence files in FASTQ format according the official base-calling pipeline. The distribution of both the Q_phred_ value-based base error rate and the GC content along reads were first investigated to verify the sequencing quality. Subsequently, we conducted quality filtering and discarded these low-quality reads, which belonged to one of the following types: (1) reads containing adaptor sequences, (2) reads containing unambiguous bases of N more than 10% of the total length, and (3) reads containing low-quality bases (Q <5) more than 50% of the total length. If any member of the paired reads was marked as low quality, both pairs were simultaneously discarded.After these steps, we obtained clean reads for the following analyses.

### SNPs calling

The Stacks tool set [13]was employed to call variants among clean reads, which is a popular method for efficiently analyzing genotype-by-sequencing data. Although the paired-end reads were sequenced in the present study, only reads from the anchored ends by restriction enzymes were subjected to variant calling, because the paired opposite reads are position-free and do not stack-up. Additionally, we employed the *de novo* algorithm for variant calling because there is currently no reference genome sequence available.

According to the official recommendation of Stacks [13], the whole analysis pipeline was first performed by the wrapped scrip (denovo_map.pl). During this process, the critical parameters were provided with the minimum number of reads required to create a stack (-m 3),the number of mismatches allowed between loci when processing a single individual (-M 2), and the number of mismatches allowed when aligning secondary reads to primary stacks (-N 4). These steps generated all raw SNPs, genotypes and haplotypes for each individual, which were subsequently corrected by the integrated population-wide data (restacks module). Therefore both modules of cstacks and sstacks were sequentially rerun on existing data; their results were further fed to the population module to finally produce the full list of SNPs at each polymorphism position and the individual genotypes and haplotypes.

### Genetic diversity and phylogenetic relationship

For these generated SNPs, we first investigated basic properties using custom scripts, such as the numbers of transition and transversion type substitutions. The nucleotide diversity at each nucleotide position and the haplotype diversity at each locus were calculated by Stacks [13]; we also analyzed the observed and expected heterozygosity. The distribution of the inbreeding coefficient of an individual relative to the subpopulation (F_IS_) was also demonstrated.To dissect the individual genetic relatedness, the pair-wise Euclidean distances among 11 samples were calculated according to all clean SNPs using the SNP RelateR package[14]. Subsequently, the calculated dissimilarities matrix was subjected to both principal coordinate analysis using Stat R packages and hierarchical clustering to reveal their genetic relationships.

To providean overview of genome properties, the GC content was also calculated by utilizing both paired reads. Among the clean reads, a total of 10, 000 paired reads were randomly selected and subjected to homologous searching against the NCBI nucleotide databaseusing the Blast tool (-a 6 -p blastn -e 1e-05). Based on the results of homologous searches, the species most related to the hog deer could be revealed to provide robust view conceming its phylogenetic position.

### Results and discussion

In the past decade, the genome sequences of eukaryotic, prokaryotic and archaea organisms have ibecome increasingly available since the wide application of high-throughput sequencing techniques [15]. Despite this increased availability, biological researchon a large number of non-model organisms is still hindered by the absence of reference genome. RAD-seq and other genotype-by-sequencing techniques can be used independent of the reference genome and powerfully provide a landscape of genome-wide variants, which contributes significantly to investigations of population genetics and ecology[12,16]. In the present study, we successfully selected the *EcoRI* enzyme to digest the genomic DNA of hog deer for high-throughput sequencing and obtained a total of 13.34 G raw data (Table 1), which ultimately produced 13.05 G clean data after quality filtering with 4.7 million reads per sample. After removing duplicate reads, a mean of 4.6 million reads for each sample remained. The results represent to the best of our knowledge, the first successful completion of the RNA-seq technique in hog deer.

**Table 1:** Sequenced reads before and after quality filtering.

Samples	Raw reads	Clean reads	Unique reads	GC Content(%)
AX1	5, 311, 042	5, 219, 540	5, 057, 552	39.35
AX10	4, 558, 485	4, 465, 887	4, 339, 728	39.85
AX11	5, 435, 037	5, 296, 912	5, 126, 204	39.25
AX12	4, 517, 627	4, 443, 334	4, 317, 832	39.49
AX14	4, 883, 124	4, 747, 291	4, 609, 039	39.45
AX3	4, 430, 537	4, 325, 088	4, 191, 454	39.06
AX4	4, 626, 111	4, 529, 531	4, 388, 225	39.14
AX5	4, 829, 317	4, 713, 200	4, 569, 181	39.21
AX6	4, 884, 514	4, 784, 704	4, 629, 554	39.13
AX7	4, 827, 450	4, 746, 192	4, 606, 088	39.34
AX8	5, 040, 976	4, 935, 299	4, 787, 899	39.30
**Average**	**4, 849, 475**	**4, 746, 089**	**4, 602, 069**	**39.32**

**Fig 2:**
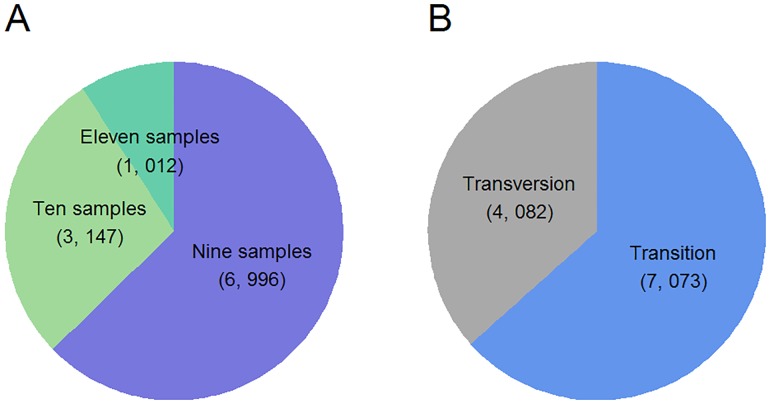
Proportions of all detected SNPs simultaneously observed among different numbers of samples (A) and the ratio of transition to transversion events(B).

Bioinformatics algorithm and analysis pipelines have been widely promoted in recent years in response to the considerably increased quantity of sequencing data. For these short reads produced from RAD-seq, many computational tools have been specifically proposed to call variants with or without dependence on the reference genome, such as RApiD[17], Stack [13,18], and PyRAD[19]. However, their respective strengths and weaknesses have not been extensively compared. According to our experience, in addition to algorithm optimization on critical steps of SNP calling, Stack also provides various functions in calculating the popular summary statistics[13]. Therefore, we employed the Stack tool for SNP calling in the initiation of clean reads, which ultimately assembled 1.40 million lociand detected 1.42 million SNPs. By setting 80% of the minimum percentage of individuals in a population to confidently support the informative locus, 143,129 loci and 11,155 SNPs finally remained. For all SNPs, 62.7%, 28.2% and 9.1% of them were present among nine, ten and eleven individuals, respectively; the relative ratio of transition to transversion events was 1.73 (Fig 2).Because more SNPs would be observed within an individual locus, a total of 8,247 haplotypes/alleles were constructed.

**Fig 3:**
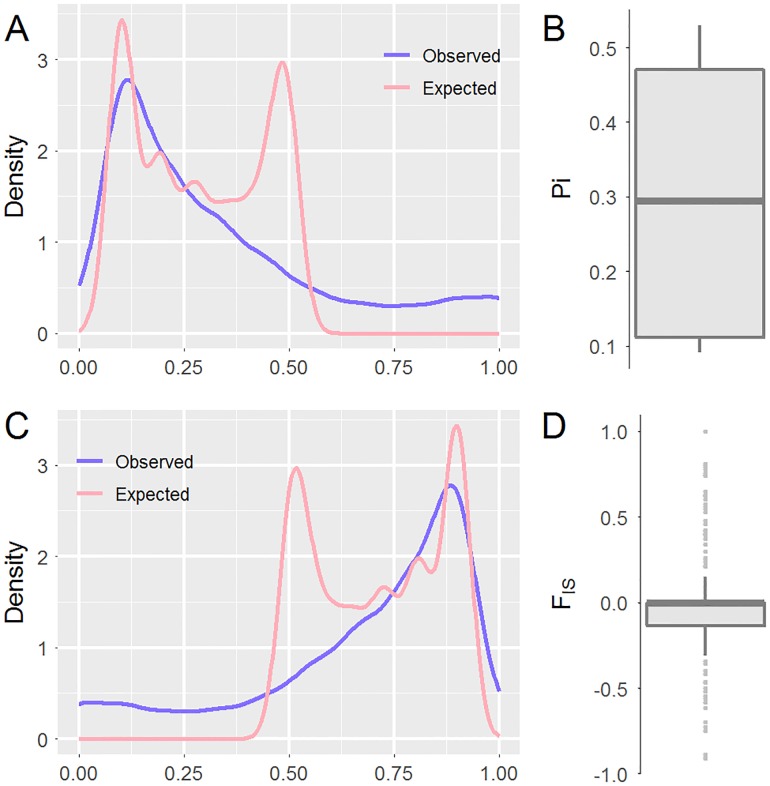
Distributions of the observed and expected heterozygosity in density (A) and box (B) plots and for the observed and expected homozygosities (C and D).

**Fig 4:**
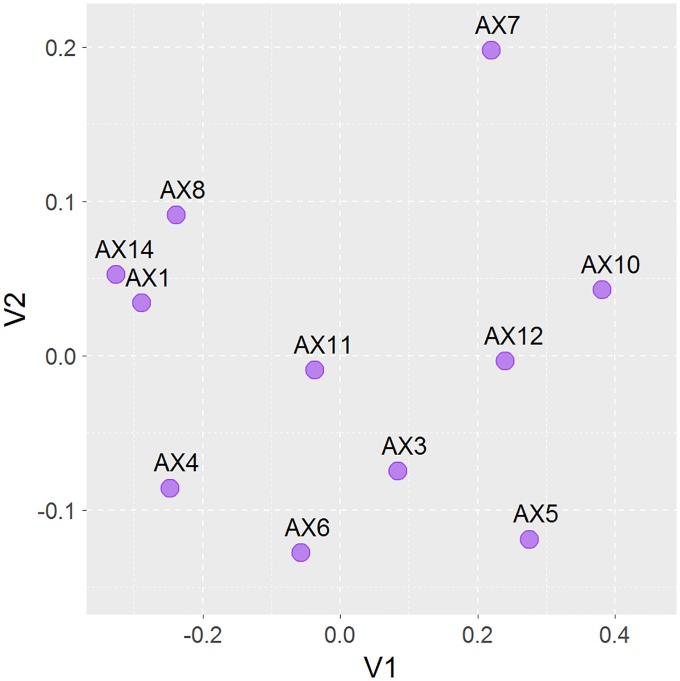
Genetic distance-based scaling plotting of pair wise comparisons according to their genetic relatedness among 11 studied hog deer.

We subsequently investigated genetic diversity at the population level based on these SNPs (Table 2). The mean frequency of the most frequent allele at each locus in this population was 0.7903±0.0014, and the mean values of nucleotide diversity (π) and inbreeding coefficient (F_IS_) were 0.3031±0.0015 and -0.0302±0.0062in the present population, respectively. The observed heterozygosity (0.3271±0.0025) was higher than expected (0.2870±0.0014). Furthermore, the density distributions of heterozygosity, homozygosity, π and F_IS_ were demonstrated among all polymorphism sites (Fig 3), revealing the overall distribution pattern. Although this study provided an overview of the genetic diversity in the captive population of hog deer reared in Chengdu Zoo, it is still impossible to perform direct comparative analysis, because little molecular data is currently available from wild and other captive populations. Lian et al. [20] developed nine novel microsatellite markers in hog deer and found average observed and expected heterozygosities of 0.397 and 0.433, respectively[20]. In another related report, microsatellite markers were employed for paternity testing of individual hog deer [21]. Additionally, based on the pair-wise Euclidean distances among these individuals, the multidimensional scaling method revealed that these 11 hog deer samples were evenly distributed (Fig 4), which was consistent with our random sampling strategy.

**Table 2:** Overview of genetic diversity in this population of 11 hog deer.

	Major allele (%)	Heterozygosity		Homozygosity		π	F_IS_
		Observed	Expected	Observed	Expected		
Mean	0.7903	0.3271	0.2870	0.6729	0.7130	0.3031	-0.0302
S.E.	0.0014	0.0025	0.0014	0.0025	0.0014	0.0015	0.0062

S.E, standard error;π, nucleotide diversity; F_IS_, the inbreeding coefficient of an individual (I) relative to the subpopulation (S).

The hog deer has long been positioned within the Axis genus based on both classical taxonomy and variations in mitochondrial and nuclear DNA sequences[2]. Alternatively, other genera, such as *Hyelaphus* and *Cervus*, have also been proposed to include hog deer [3]. However, the exact taxonomy of hog deer should be specifically investigated using more information such as mitochondrial or even nuclear genome sequences. In the present study, the average GC content of the hog deer genome was 39.2% (Table 1). Additionally, we obtained a large quantity of short paired reads 125 bp in length, which would be randomly derived from the genome. Herein, we intended to reveal the phylogenetic relationship of hog deer by blasting these reads to the NCBI nucleotide database and identifying the closest species; the top three hits of the homologous search were *Bostaurus*, *Muntiacusmuntjak*, and *Ovisaries*. Unfortunately, all three target species were further away from hog deer than those of previous reports. One possible explanation for these results is that the reference sequences of Cervinae species areincomplete. Accordingly, we believe that a homologous search based on the analysis of short reads would be useless for phylogenetic analysis. Therefore, the taxonomy of hog deer should be investigated in future, such as sequencing the entire mitochondrial genome.

## Conclusion

In the present study, we successfully employed the RAD-seq technique to generate a large quantity of SNPs at the genome level for the endangered hog deer species. Subsequent analyses also revealed relatively abundant genetic diversity preservedin this captive population. These genome-wide SNPs are expected to be used for producing the molecular maker-based mating programs to effectively avoid sharp increase of inbreeding coefficient. Of course, we failed to provide positive clue about the phylogeny of hog deer which should be addressed in future.
